# Mushroom polysaccharides from *Grifola frondosa* (Dicks.) Gray and *Inonotus obliquus* (Fr.) Pilat ameliorated dextran sulfate sodium-induced colitis in mice by global modulation of systemic metabolism and the gut microbiota

**DOI:** 10.3389/fphar.2023.1172963

**Published:** 2023-06-07

**Authors:** Runbin Sun, Dandan Jin, Fei Fei, Zhi Xu, Bei Cao, Juan Li

**Affiliations:** ^1^ Phase I Clinical Trials Unit, Nanjing Drum Tower Hospital, Affiliated Hospital of Medical School, Nanjing University, Nanjing, China; ^2^ Nanjing Drum Tower Hospital Clinical College of Nanjing University of Chinese Medicine, Nanjing, China

**Keywords:** *Grifola frondosa* polysaccharides, *Inonotus obliquus* polysaccharides, colitis, gut microbiota, metabolomics

## Abstract

**Introduction:** Polysaccharides from *Grifola frondosa* (Dicks.) Gray (HSH) and *Inonotus obliquus* (Fr.) Pilat (BHR) showed noticeable effects on dextran sulfate sodium (DSS)-induced colitis, but their systemic modulation effects have not been fully revealed. This study aimed to investigate the regulation of the gut microbiota and systemic metabolism by HSH and BHR in DSS-induced colitis.

**Methods:** C57BL/6J mice were given DSS (2.5%) in water and were treated with HSH and BHR (200 mg/kg/day) by gavage. Body weight and colon length were recorded, and H&E and AB-PAS staining of the colon were conducted to evaluate the model and the protective effect of the polysaccharides. Additionally, an LC-QTOF/MS-based untargeted metabolomic platform was used to identify the metabolites in the serum, colon tissue, gut contents, and faeces and investigate differential metabolites and metabolic pathways. 16S rDNA gene sequencing was used to measure the composition of bacterial communities.

**Results:** The results showed that the mouse colitis model was established successfully, as evidenced by an increased disease activity index score [2.83 ± 0.62 vs. 0.06 ± 0.14 (*p* < 0.001)] and shortened colon length [5.43 ± 0.64 cm vs. 7.04 ± 0.29 cm (*p* < 0.001)], and HSH and BHR ameliorated DSS-induced colitis by improving the disease activity index (2.17 ± 0.28 and 1.83 ± 0.29, respectively) and restoring the colon length (6.12 ± 0.30 cm and 6.62 ± 0.35 cm, respectively). HSH and BHR significantly modulated metabolites involved in aromatic amino acid metabolism, the citrate cycle, purine metabolism, pyrimidine metabolism, etc. HSH and BHR increased the Chao1 index by 64.25% and 60.25%, respectively, and they increased the Shannon index by 13.02% and 10.23%, respectively. They both reversed the increase in the abundances of *g_Odoribacter*, *g_Clostridium*, *g_AF12*, *g_Parabacteroides* and *g_Turicibacter* and reversed the decrease in the abundance of *g_unclassified_Bacteria* induced by DSS. Specifically, HSH reversed the reductions in *g_unclassified_Lactobacillales* and *g_Ruminococcus*, and BHR reversed the decreases in *g_unidentified_Coriobacteriaceae* and *g_unclassified_Firmicutes*.

**Discussion:** These results suggested that HSH and BHR may ameliorate DSS-induced colitis by global modulation of systemic metabolism and the gut microbiota. Targeting the gut microbiota may be a potentially effective strategy to modulate systemic metabolism and treat colitis.

## 1 Introduction

Ulcerative colitis (UC) is a chronic colorectal mucosal inflammatory disease caused by multiple factors. In recent years, its incidence and prevalence have been increasing worldwide. UC often begins in the rectum, the symptoms gradually worsen, and the lesion area extends to the colon and even the ileocecal region (Ordás et al., 2012). The disease is characterized by long course of recurrent attacks, remission, delayed healing and non-specificnonspecific ulcers. The typical clinical manifestations are abdominal pain, diarrhoea and mucopurulent bloody stool. At present, the pathogenesis of UC is still in the exploratory stage. However, psychological factors, genetic factors, environmental factors, intestinal mucosal barrier function and other factors play important roles in the pathogenesis of UC, which can lead to mucosal barrier damage, thereby causing local ulcers of the intestinal mucosa (Ungaro et al., 2017). Ulcerative colitis poses severe medical and financial stress (Kaplan, 2015). Studies have shown that its incidence is significantly related to age, social environment, dietary habits, geographical location and ethnic background (Logan and Bowlus, 2010; Molodecky et al., 2012). Several studies have investigated the association between diet and the potential risk of UC: the consumption of red meat, fats, and sweets is positively associated with the development of UC (Hansen et al., 2011; Hou et al., 2011; Ananthakrishnan et al., 2014), and breastfeeding is negatively associated with the risk of subsequent UC (Xu et al., 2017).

The gut microbiota is a general term for various microbial communities that reside in the host’s gut and coexist with the host, including bacteria, archaea and fungi (Lynch and Pedersen, 2016). Under normal physiological conditions, good bacteria, harmful bacteria, and pathogens in the intestinal flora maintain a dynamic balance. Once the balance is broken, it can lead to alterations in the intestinal flora diversity and structure and changes in bacterial abundance, especially reducing probiotics and increasing pathogens, which will cause some diseases. Intestinal flora imbalance is therefore the potential pathogenesis of UC (Pei et al., 2019). In response to an imbalance in the intestinal flora, a wide variety of bioactive metabolites and toxins are produced, which enter various body organs via the blood. As a result, metabolomics technology can be used to identify the specific compounds linked to the disease that are present in faeces, blood, urine, and tissue fluid, allowing the analysis of the host phenotype, microbiome, and metabolomic data to reveal potential connections associated with the mechanism. The combination of sequencing technology and metabolic technology will further explore the changes in nonbacterial organisms and metabolites of the intestinal flora and more accurately explain the mechanism of protecting against UC by regulating the intestinal flora (Li W. et al., 2021).

The pathogenesis of UC is complex, and the aetiology is still unclear. Currently, the drugs for treating UC mainly include 5-aminosalicylates, glucocorticoids, immunosuppressants, and interferon (Akiho et al., 2015). Although these drugs produce apparent efficacy, they cannot completely cure UC. Long-term use of these drugs will increase the economic burden on patients. Additionally, drug use can cause different adverse reactions and toxic side effects, such as abdominal pain, kidney injury, liver toxicity and blood diseases. Hence, the development of medical therapy needs to be further explored. Traditional Chinese medicine (TCM) prescriptions have a long history in treating UC, and similar symptoms of UC were recorded in ancient China. According to the theory of traditional Chinese medicine, the clinical treatment of UC is primarily to clear heat, remove dampness, stop dysentery, and strengthen the spleen and kidney in accordance with the typical clinical signs and characteristics of abdominal pain, diarrhoea, mucopurulent bloody stool, and tenesmus in UC. In conclusion, TCM theory posits that the occurrence of UC is closely related to the liver, spleen and kidney and is also affected by case factors such as dampness, cold and heat (Huang et al., 2019; Zhang X. et al., 2022). Chinese herbal medicine has become a research hotspot in recent years due to its multicomponent, multitarget, multipathway, and holistic regulation characteristics. Under the guidance of traditional Chinese medicine theory, the use of classic prescriptions in the treatment of UC has attracted the attention of a large number of researchers and allowed significant research progress. Studies have shown that Chinese herbal medicine can effectively improve the symptoms of UC and has shown unique advantages in the prevention and treatment process, such as multitarget regulation, precise efficacy, minor side effects, and low recurrence rates.

Polysaccharides are essential active ingredients of traditional Chinese medicine, and they have been reported as the material basis of the drug effects in UC treatment. Polysaccharides are polymeric macromolecular carbohydrates composed of more than ten monosaccharides that are widely found in many Chinese medicinal materials, such as *Panax ginseng* C. A. Meyer, *Ganoderma lucidum* (Leyss. Ex Fr.) Karst, *Astragalus membranaceus* (Fisch.) Bge, *Lycium barbarum* Linn., and *Lentinus edodes* (Berk.) Sing. Polysaccharides have various biological activities, such as antitumour, antioxidation, antiviral, immunomodulation, lipid-lowering, and neuroprotection activities (Feng et al., 2019; Guo et al., 2021). It has been reported that polysaccharides can effectively improve the symptoms of UC, mainly by protecting the intestinal mucosal barrier (upregulating tight junction proteins, decreasing the permeability of the intestinal mucosa, regulating intestinal mucus function, protecting intestinal epithelial cells and reducing oxidative stress) and regulating the intestinal mucosal immune response (regulating intestinal mucosal immune cells, immune cytokines and intestinal mucosal signalling pathways, including the JAK-STAT, NF-κВ, MAPK and PI3K/Akt/mTOR signalling pathways) (Wang et al., 2022). Regulating the intestinal flora is another important potential target for treating UC by Chinese herbal medicine (Huang et al., 2019; Zhang X. et al., 2022). An increasing number of studies have shown that the polysaccharide components of some traditional Chinese medicines also have the function of intestinal flora regulation, thereby changing the body’s metabolism and improving colon tissue damage. Therefore, the intestinal flora may also be a potential target of the polysaccharide components of traditional Chinese medicines.

*Grifola frondosa* (Dicks.) Gray, a full botanical name authorized by “Index Fungorum” (http://www.indexfungorum.org.), belongs to the family Meripilaceae. Its local name is Maitake mushroom. It is a rare edible and medicinal fungus that developed in recent years and is mainly distributed in China, Russia and Japan. *Inonotus obliquus* (Fr.) Pilat (local name Chaga mushroom, belonging to the family Hymenochaetaceae), is a precious fungus with both medicinal and food uses. *I. obliquus* is mainly distributed in Russia, northeast China, Japan, and other places from 45° to 50° north latitude. *G. frondosa* polysaccharide (HSH) and *I. obliquus* polysaccharide (BHR), as natural plant medicines, have a wide range of pharmacological effects. Their most significant therapeutic value lies in their hypoglycaemic effect, immunomodulatory effect, antitumour effect, antioxidant effect and so on (Adachi et al., 1994; Kurushima et al., 2000; Lin, 2011; Balandaykin and Zmitrovich, 2015; Glamočlija et al., 2015). The wide range of biological activities and nontoxic effects of the polysaccharides of *G. frondosa* and *I. obliquus* provide them with excellent potential for clinical application. These polysaccharides were both notably effective for treating DSS-induced colitis. However, the interaction mechanisms between HSH and BHR with target cells and the whole body have not yet been fully elucidated, so further research is needed to reveal the specific therapeutic targets and their therapeutic mechanisms. To explore the relationship between HSH and BHR with the gut microbiota and the underlying mechanisms for treating DSS-induced colitis, an experiment in mice was conducted to evaluate the variations in inflammatory cytokines and uncover the changes in metabolites in serum, colon tissue, the gut contents and faeces, as well as the overall changes in the gut microbiota. This study investigated the regulation of the gut microbiota and systemic metabolism by HSH and BHR in DSS-induced colitis. This research may provide new mechanisms and potential targets for colitis therapy involving the intestinal ecosystem.

## 2 Methods

### 2.1 Materials

Dextran sulfate sodium (DSS, MW: 36–50 kDa) was purchased from MP Biochemicals (Santa Ana, CA, United States). 4-Chlorophenylalanine was purchased from Aladdin Biochemical Technology Co., Ltd. (Shanghai, China). Methanol and acetonitrile were purchased from Merck KGaA (Darmstadt, Germany). Polysaccharides from *G. frondosa* (69.13%, Mw 10.43 KDa, Glucose, Mannose, Galactose and Glucosamine in molar ratios of 55.0:4.9:3.0:1.9) and *I. obliquus* (74.70%, 17.08 KDa, Glucose, Mannose, Galacturonic Acid, Arabinose and Rhamnose in molar ratios of 40.7:8.5:2.7:2.1:0.2) were provided and identified by Xi’an Tongze Biotechnology Co., Ltd. (Xi’an, China).

### 2.2 Animals and treatment

24 Male C57BL/6J mice (5–6 weeks old) were purchased from Changzhou Cavens Experimental Animal Co., Ltd. (Changzhou, China). Mice were acclimatized under 12 h/12 h dark-light cycles at a constant temperature (22°C ± 2°C) and had free access to water and food. All animal care and experimental procedures protocols were approved by the Animal Ethics Committee of Nanjing University Medical School Affiliated Drum Tower Hospital. All mice were fed a normal diet for 1 week to acclimate to the environment. After that, they were divided into four groups (n = 6), the Control group, the DSS group, DSS + HSH (*G. frondosa* Polysaccharide) group and DSS + BHR (*I. obliquus* Polysaccharide) group. For the first 3 days, mice in the DSS + HSH group and DSS + BHR group were intragastrically treated with *G. frondosa* Polysaccharide (200 mg/kg, dissolved in 0.5% CMCNa) and *I. obliquus* Polysaccharide (200 mg/kg, dissolved in 0.5% CMCNa) every day, while mice in the Control group were given Control solution (0.5% CMCNa) by gavage. After that, mice drank sterilized water with (DSS group, DSS + HSH group and DSS + BHR group) or without (Control group) 2.5% DSS solution for 1 week. Meanwhile, mice in the DSS + HSH group and DSS + BHR group were intragastrically treated with *G. frondosa* Polysaccharides (200 mg/kg, dissolved in 0.5% cyclodextrin) and *I. obliquus* Polysaccharide (200 mg/kg, dissolved in 0.5% CMCNa) every day during the week, and mice in the Control group were given Control solution (0.5% CMCNa) by gavage. The detailed process of animal administration is shown in Supplementary Figure S1. After the treatment, mice were euthanized by avertin, and serum, colon, gut content and faeces were collected and kept at −80°C for further analysis.

### 2.3 Sample preparation and LC-QTOF/MS analysis

The same extraction and analysis procedures were applied as previously reported with a few modifications. In brief, 200 μL of methanol containing 1 μg/mL of 4-Chlorophenylalanine as an internal standard (IS) was added to 50 μL of serum, and 20 mg of colon tissue was homogenized with 200 μL of 80% methanol containing 1 μg/mL of 4-Chlorophenylalanine, about 50 mg of gut content and faeces was homogenized with 500 μL of 80% methanol containing 1 μg/mL of 4-Chlorophenylalanine to extract the metabolites and precipitate the protein. After that, 200 μL of supernatant was transferred to a new Eppendorf tube and evaporated to dryness on an SPD 2010–230 SpeedVac Concentrator (Thermo Savant, Holbrook, United States). The residue was re-dissolved with 100 μL distilled water and centrifuged at 18,000 rpm for 10 min. Finally, 80 μL supernatant was transferred to an LC vial and 5 μL was injected into an ACQUITY UPLC@HSS T3 HPLC column (1.8 µm; 2.1 mm × 100 mm; Waters, United States). The column temperature for analysis was set to 40°C. The LC-QTOF/MS consisted of an LC system (ExionLC, Foster City, CA) coupled with a hybrid quadrupole time-of-flight tandem mass spectrometer (AB SCIEX TripleTOF^®^ 5600 LC-QTOF/MS, Foster City, CA). The mobile phase consisted of solvent A (water with 0.1% formic acid) and solvent B (acetonitrile with 0.1% formic acid) with the following gradient: 0–1.5 min 5% B, 1.5–2.5 min 5%–15% B, 2.5–6 min 15%–60% B, 6–10 min 60%–95% B, 10–12 min 95% B, 12–12.5 min 95%–5% B, 12.5–15.5 min 5% B. The flow rate was 0.4 mL/min. The mass detection was performed in positive (4500 V) and negative (−5500 V) ion modes for scan analysis with Turbo V electrospray ionization (ESI). The parameters were set as follows: ion spray voltage, 7 kV; turbo spray temperature (TEM), 550°C; declustering potential (DP), 70 V; collision energy (CE), 35 eV; nebulizer gas (gas1), 55 psi; heater gas (gas 2), 55 psi; curtain gas, 35. The nebulizer and auxiliary gas were kept by Nitrogen. TOF MS ranged from m/z 50–1,200. Automatic calibration was carried out every twenty samples.

### 2.4 Compound identification

MS-DIAL 3.5.2 (http://prime.psc.riken.jp) was used for peak detection, spectral deconvolution, peak alignment and compound identification. Databases including HMDB (https://hmdb.ca/), METLIN (http://metlin.scripps.edu), and an in-house database established in our lab were used to identify and interpret LC-QTOF/MS chromatographic peaks. The metabolites were identified by comparing the detected mass spectra and retention time of the compounds.

### 2.5 Multivariate and univariate data analysis

Peak areas of detected compounds were normalized by the area of IS for each sample. Principal components analysis (PCA) and partial least squares discriminant analysis (PLS-DA) was performed using the mixOmics package in the R project (version 4.2.1). The PCA model was used to see the overall distribution of all the samples, and the PLS-DA model was used to confirm the general separation among the four groups. Variable of importance in the project (VIP) analysis was used to identify the endogenous metabolites contributing to the classification. Furthermore, univariate analysis was conducted by one-way analysis of variance (ANOVA) followed by a pairwise *t*-test and corrected by the Benjamini–Hochberg method to control the False Discovery Rate (FDR). Peaks that had a *p*-value less than 0.05 were considered to be differential. The differential metabolites selected by multivariate and univariate data analysis were further analyzed by fold change among the four groups.

### 2.6 Pathway analysis

Metabolomics pathway analysis of the differential metabolites was carried out using the online metabolomics data analysis tool MetaboAnalyst 5.0 (www.metaboanalyst.ca) with the KEGG library (*Mus musculus*).

### 2.7 16S rDNA gene sequencing

Genomic DNA from the gut content of mice in each group was extracted following the protocol of Fast DNA™ SPIN Kit (MP Biomedicals, CA, United States). Briefly, about 100 mg of mouse gut content sample was weighed and the total DNA was extracted and quantified by Nanodrop. The target fragment was amplificated by high-fidelity PCR. Then the amplified products were purified and recovered with magnetic beads. The recovered products of PCR amplification were quantified by fluorescence with the fluorescent reagent Quant-iT PicoGreen dsDNA Assay Kit, and the quantitative instrument was a Microplate reader (BioTek, FLx800). According to the quantitative fluorescence results, the samples were mixed according to the corresponding proportion according to the sequencing volume requirement of each sample. The sequencing library was prepared with TruSeq Nano DNA LT Library Prep Kit (Illumina, San Diego, CA, United States). The quality inspection of the sequencing library was performed on the Agilent Bioanalyzer using the Agilent High Sensitivity DNA Kit. The library was quantified using the Quant-iT PicoGreen dsDNA Assay Kit on the Promega QuantiFluor fluorescence quantification system. The 16S rDNA gene amplicon sequencing was performed on an Illumina MiSeq platform (Illumina, San Diego, CA, United States) by Suzhou PANOMIX Biomedical Tech Co. Ltd. (Suzhou, China).

### 2.8 Statistical analysis

GraphPad Prism 7.2 software (La Jolla, CA, United States) and R project (version 4.2.1) were adopted for statistical analysis. Data were presented as mean ± standard deviation. Differences among groups were analyzed by a one-way ANOVA followed by the Bonferroni *post hoc* test. For all statistical tests, *p* < 0.05 was considered significant.

## 3 Results

### 3.1 Polysaccharides from *G. frondosa* and *I. obliquus* ameliorated DSS-induced colitis in mice

**FIGURE 1:**
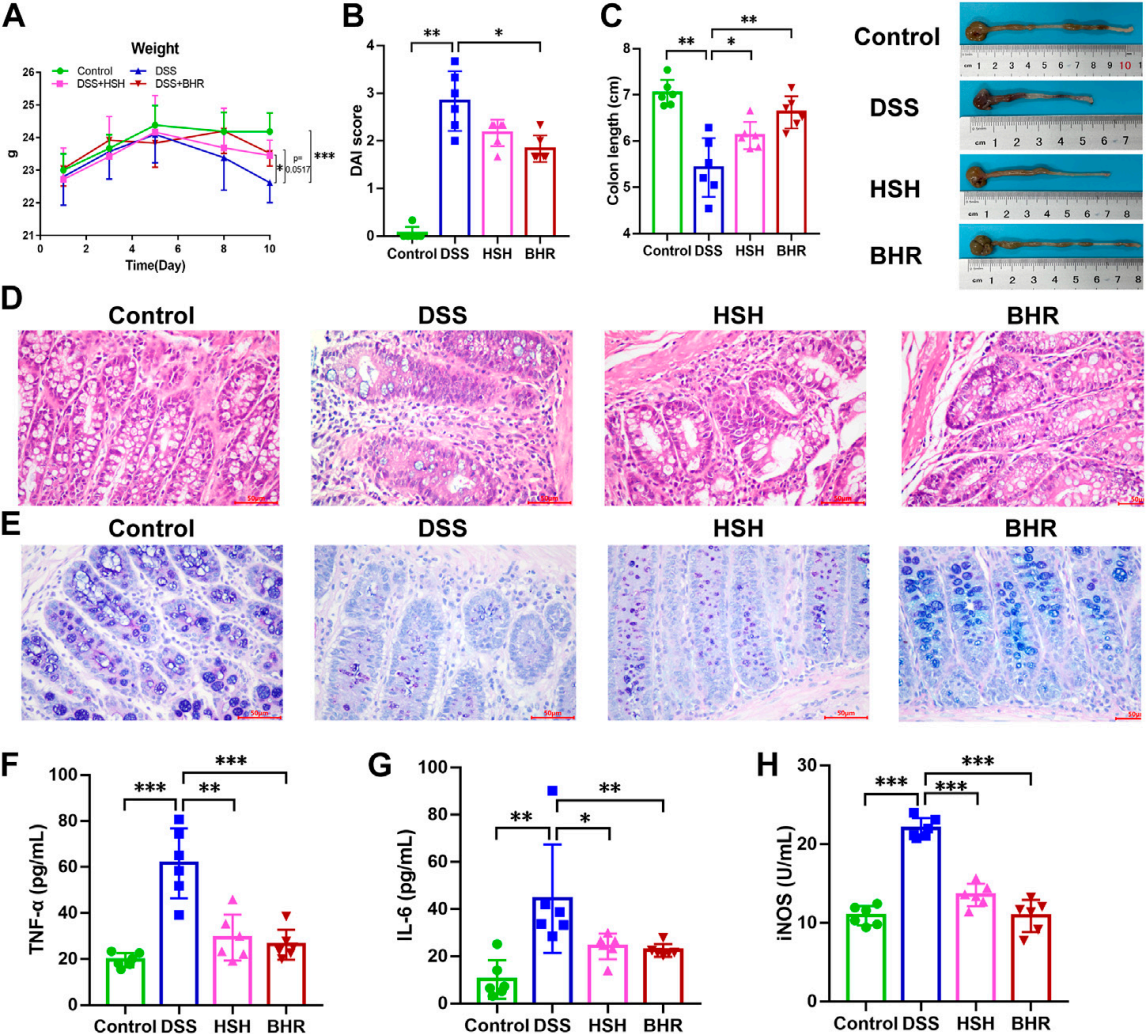
Ameliorative effect of HSH and BHR on DSS-induced colitis in mice. **(A)** Body weight change of C57BL/6J mice after treatment with HSH and BHR. **(B)** DAI score of C57BL/6J mice after treatment with HSH and BHR. **(C)** Colon length of C57BL/6J mice after treatment with HSH and BHR for 7 days. **(D)** Representative H&E-stained colon tissues (×200 magnification) in C57BL/6J mice after treatment with HSH and BHR. **(E)** Representative AB PAS-stained colon tissues (×200 magnification) in C57BL/6J mice after treatment with HSH and BHR. **(F)** Levels of TNF-α in serum. **(G)** Levels of IL-6 in serum. **(H)** The serum iNOS activity. Data are shown as mean ± SD (n = 6). *p < 0.05, **p < 0.01, ***p < 0.001.

We first evaluated the effect of polysaccharides from *G. frondosa* and *I. obliquus* on DSS-induced colitis in mice. After DSS treatment for 7 days, the mice in the DSS group exhibited typical symptoms of colitis, including diarrhoea, haematochezia, and weight loss. As indicated in Figure 1A, we observed a continuous decrease in the body weight of DSS-treated mice (*p* < 0.05, DSS group vs. control group), a higher disease activity index (DAI) score (*p* < 0.01, DSS group vs. control group) (Figure 1B) and shortened colon length (*p* < 0.01, DSS group vs. control group) (Figure 1C), and HSH and BHR inhibited the reduction in body weight (*p* = 0.0517, HSH group vs. DSS group, and *p* < 0.001, BHR group vs. DSS group) (Figure 1A) and colon length (*p* < 0.05, HSH group vs. DSS group, and *p* < 0.01, BHR group vs. DSS group) (Figure 1C). BHR decreased the DAI score (*p* < 0.05, BHR group vs. DSS group) (Figure 1B). The histochemical results indicated that HSH and BHR alleviated damage to the villous membrane and increased the thickness of muscle layers in the colon tissues of DSS-treated mice (Figures 1D, E), as evidenced by restored histopathological staining. HSH and BHR also decreased the elevated levels of TNF-α, IL-6 and iNOS activity in the serum caused by DSS treatment (Figures 1F–H). From the above results, HSH and BHR significantly improved colitis-related symptoms in DSS-treated mice.

### 3.2 Overview of the metabolomic profile of the serum, colon, gut contents and faeces

The representative total ion current (TIC) chromatograms of mouse serum, colon, gut contents and faeces in positive and negative modes are shown in Supplementary Figures S2, S3. In serum, 10,969 and 12,898 chromatographic features were deconvoluted and extracted in positive mode and negative mode, respectively. In the colon, 14,575 and 10,315 chromatographic features were deconvoluted and extracted in positive mode and negative mode, respectively. In the gut contents, 21,929 and 23,406 chromatographic features were deconvoluted and extracted in positive mode and negative mode, respectively. In faeces, 22,555 and 21,592 chromatographic features were deconvoluted and extracted in positive mode and negative mode, respectively.

### 3.3 Polysaccharides from *G. frondosa* and *I. obliquus* affect metabolites in DSS-treated mice

**FIGURE 2:**
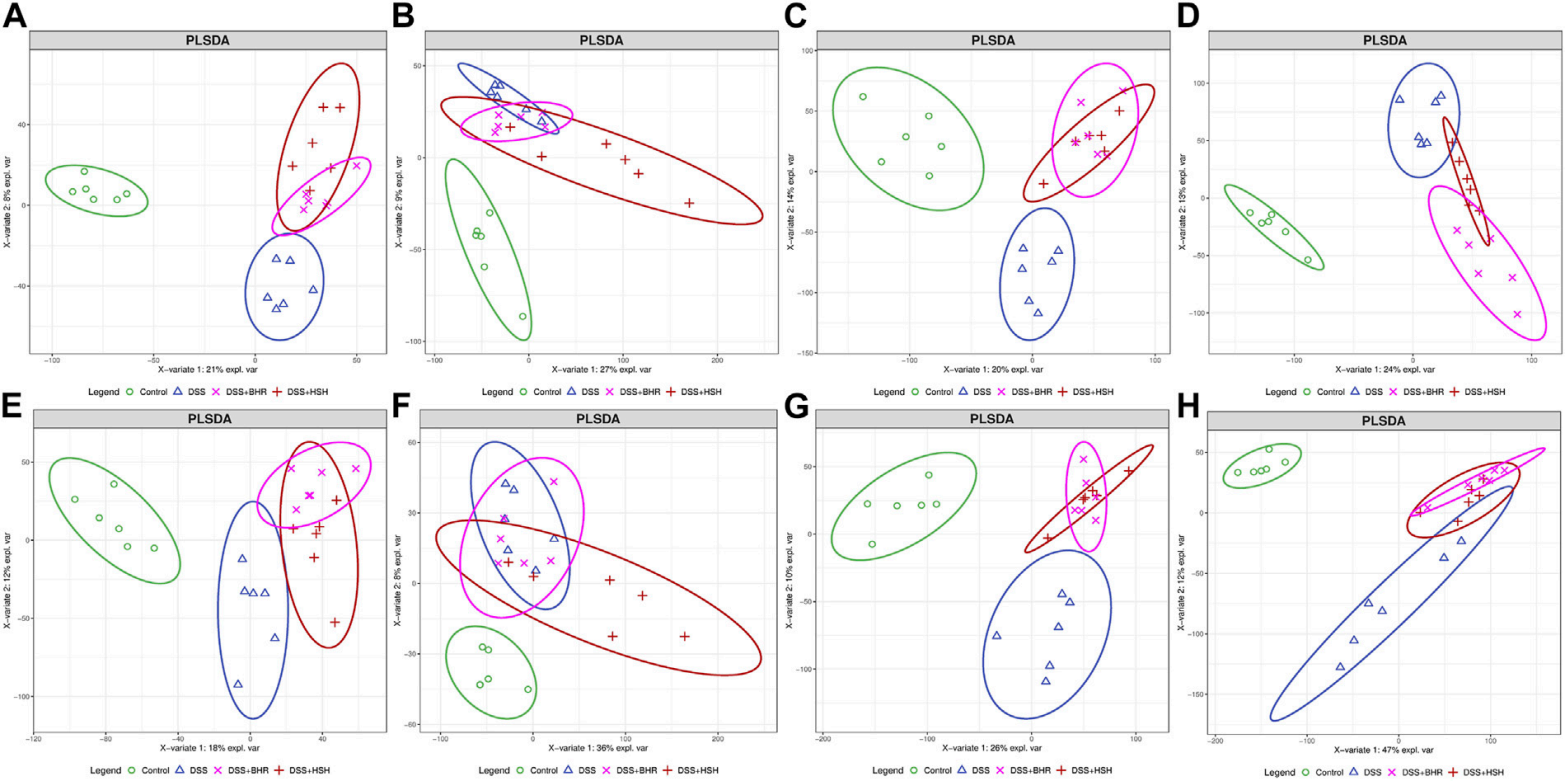
The overall modulation effect of HSH and BHR on DSS-induced colitis mouse model. Scores plots of positive mode **(A)** and negative mode **(B)** of PLS-DA model of mouse serum from 4 groups. Scores plots of positive mode **(C)** and negative mode **(D)** of PLS-DA model of mouse colon from 4 groups. Scores plots of positive mode **(E)** and negative mode **(F)** of PLS-DA model of mouse gut content from 4 groups. Scores plots of positive mode **(G)** and negative mode **(H)** of PLS-DA model of mouse faeces from 4 groups.

The acquired areas of each peak in the serum, colon, gut contents and faeces were normalized by the area of IS and analysed by PCA and PLS-DA models to obtain an overview of the metabolic variations. In the PCA model, QC samples were gathered together, suggesting that the experiments were stable and reliable (Supplementary Figures S4A–H). For serum samples, PLS-DA models of metabolites in the positive and negative modes were established (Figures 2A, B). A good separation of the control group and DSS group was observed, suggesting that the metabolic profile of the DSS-treated mice was quite different from the profile of the control group. The dots representing the HSH and BHR groups all moved away from the control group and DSS group, suggesting an apparent modulation of metabolism by HSH and BHR. Similarly, PLS-DA models of colon, gut content and faeces samples were established (Figures 2C–H), and a good separation of the control group and DSS group was also observed. The HSH and BHR groups also moved away from the DSS group, suggesting an apparent modulation in the metabolism of the colon, gut contents and faeces by HSH and BHR.

To select the differential compounds related to colitis progression and HSH and BHR treatment, the fold change (FC) was calculated by dividing the average of every two groups, and the *p* value was calculated by Student’s t test corrected by the Benjamini‒Hochberg method. The volcano plot shows metabolites with an FC of more than 2 or less than 0.5 and a *p* value of less than 0.05 in the serum (Supplementary Figures S5A–F), colon (Supplementary Figures S6A–F), gut contents (Supplementary Figures S7A–F) and faeces (Supplementary Figures S8A–F). The heatmaps of differential metabolites in the serum, colon, gut contents and faeces of the positive and negative modes are shown in Supplementary Figures S5G, H, Supplementary Figures S6G, H, Supplementary Figures S7G, H and Supplementary Figures S8G, H.

### 3.4 Mushroom polysaccharides from *G. frondosa* and *I. obliquus* significantly changed the metabolic pathways in DSS-treated mice

The significantly changed metabolites in serum, colon, gut content and faeces samples were selected, and these metabolites were further analysed by the online metabolic pathway analysis tool MetaboAnalyst (http://www.metaboanalyst.ca). The differential metabolites were mapped to KEGG metabolic pathways for overrepresentation and pathway analyses in the serum, colon, gut contents and faeces. The pathway impact factor was evaluated by the relative importance of the metabolites. A pathway was considered significantly related if it had an impact value higher than 0.05 and a *p* value less than 0.05.

**FIGURE 3:**
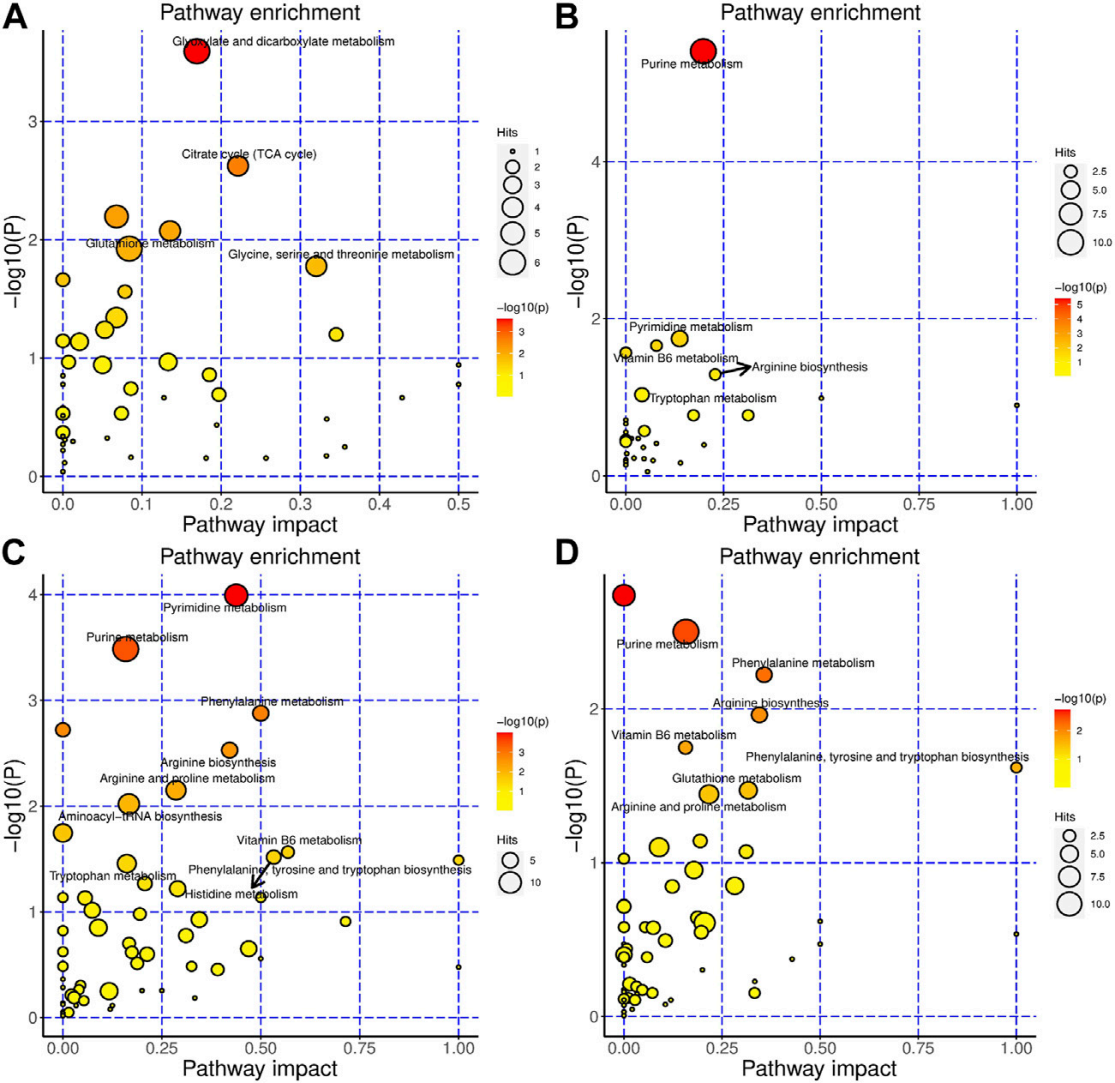
HSH significantly changed the metabolism of the DSS-induced colitis mouse model. Metabolic pathways that are significantly modulated by HSH of serum **(A)**, colon **(B)**, gut content **(C)** and faeces **(D)**, respectively.

For HSH, in serum, glyoxylate and dicarboxylate metabolism, citrate cycle (TCA cycle), tryptophan metabolism, glutathione metabolism, and purine metabolism were the most changed pathways (Figure 3A). Purine metabolism, pyrimidine metabolism, vitamin B6 metabolism, and ascorbate and aldarate metabolism were the most altered pathways in the colon (Figure 3B). In the gut contents, purine metabolism; pyrimidine metabolism; phenylalanine metabolism; arginine biosynthesis; arginine and proline metabolism; vitamin B6 metabolism; histidine metabolism; phenylalanine, tyrosine and tryptophan biosynthesis; and tryptophan metabolism were the most changed pathways (Figure 3C). In faeces, purine metabolism; phenylalanine metabolism; arginine biosynthesis; vitamin B6 metabolism; phenylalanine, tyrosine and tryptophan biosynthesis; glutathione metabolism; and arginine and proline metabolism were the most changed pathways (Figure 3D).

**FIGURE 4:**
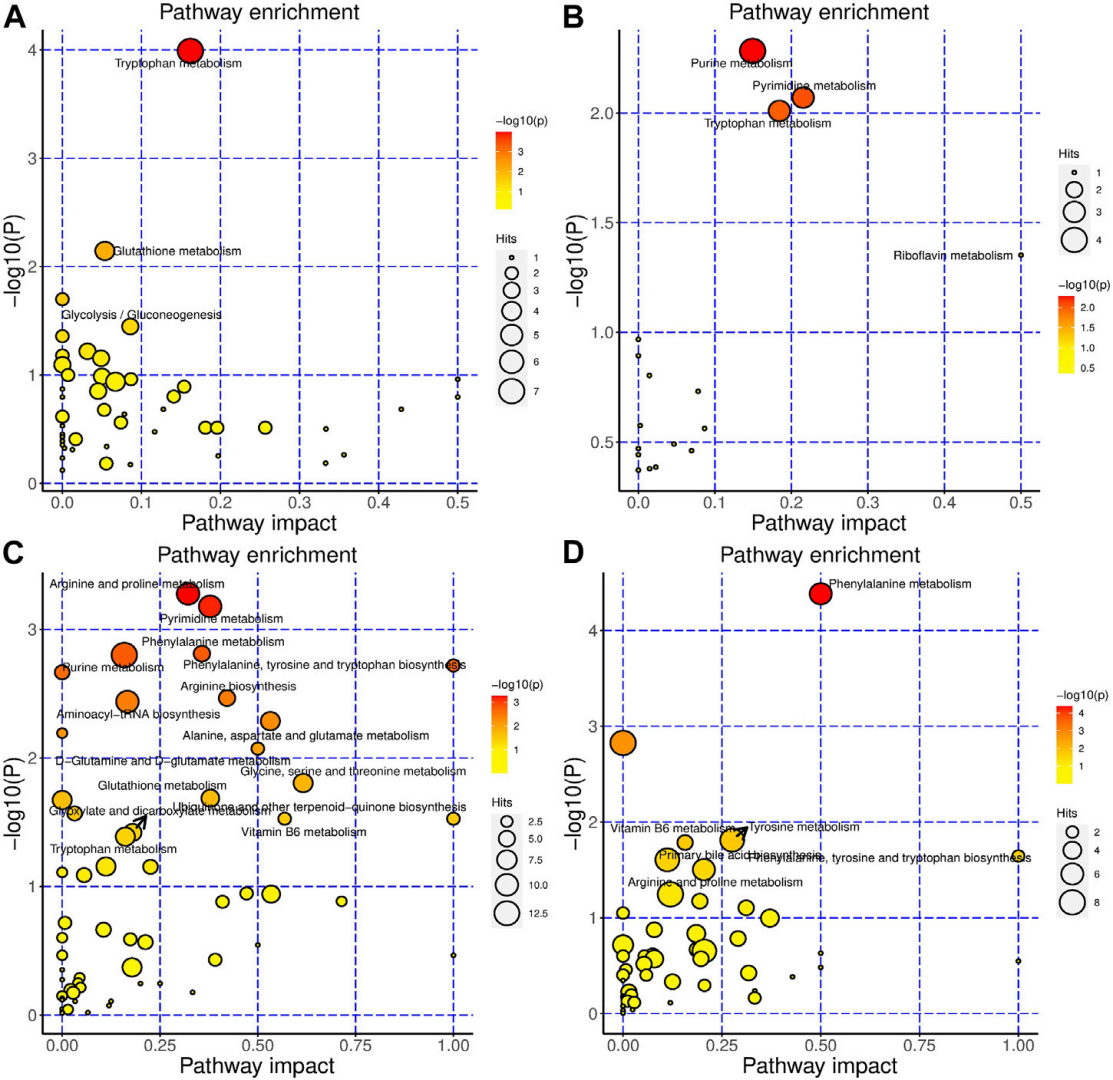
BHR significantly changed the metabolism of the DSS-induced colitis mouse model. Metabolic pathways that are significantly modulated by BHR of serum **(A)**, colon **(B)**, gut content **(C)** and faeces **(D)**, respectively.

For BHR, in serum, tryptophan metabolism, glutathione metabolism, glycolysis/gluconeogenesis, glyoxylate and dicarboxylate metabolism were the most changed pathways (Figure 4A). Purine metabolism, pyrimidine metabolism, tryptophan metabolism, and riboflavin metabolism were the most altered pathways in the colon (Figure 4B). In the gut contents, arginine and proline metabolism; pyrimidine metabolism; phenylalanine metabolism; purine metabolism; phenylalanine, tyrosine and tryptophan biosynthesis; arginine biosynthesis; alanine, aspartate and glutamate metabolism; D-glutamine and D-glutamate metabolism; glycine, serine and threonine metabolism; glutathione metabolism; vitamin B6 metabolism; ubiquinone and other terpenoid-quinone biosynthesis; glyoxylate and dicarboxylate metabolism; and tryptophan metabolism were the most changed pathways (Figure 4C). In faeces, phenylalanine metabolism; tyrosine metabolism; vitamin B6 metabolism; phenylalanine, tyrosine and tryptophan biosynthesis; primary bile acid biosynthesis; and arginine and proline metabolism were the most changed pathways (Figure 4D).

Taken together, AAA metabolism, citrate cycle (TCA cycle), purine metabolism, and pyrimidine metabolism were the most significantly changed pathways influenced by both HSH and BHR (Supplementary Figures S9A, B). Specifically, HSH modulated carbohydrate metabolism more obviously, while BHR modulated tryptophan metabolism.

### 3.5 Polysaccharides from *G. frondosa* and *I. obliquus* rebalance the structure of the gut microbiota in DSS-treated mice

**FIGURE 5:**
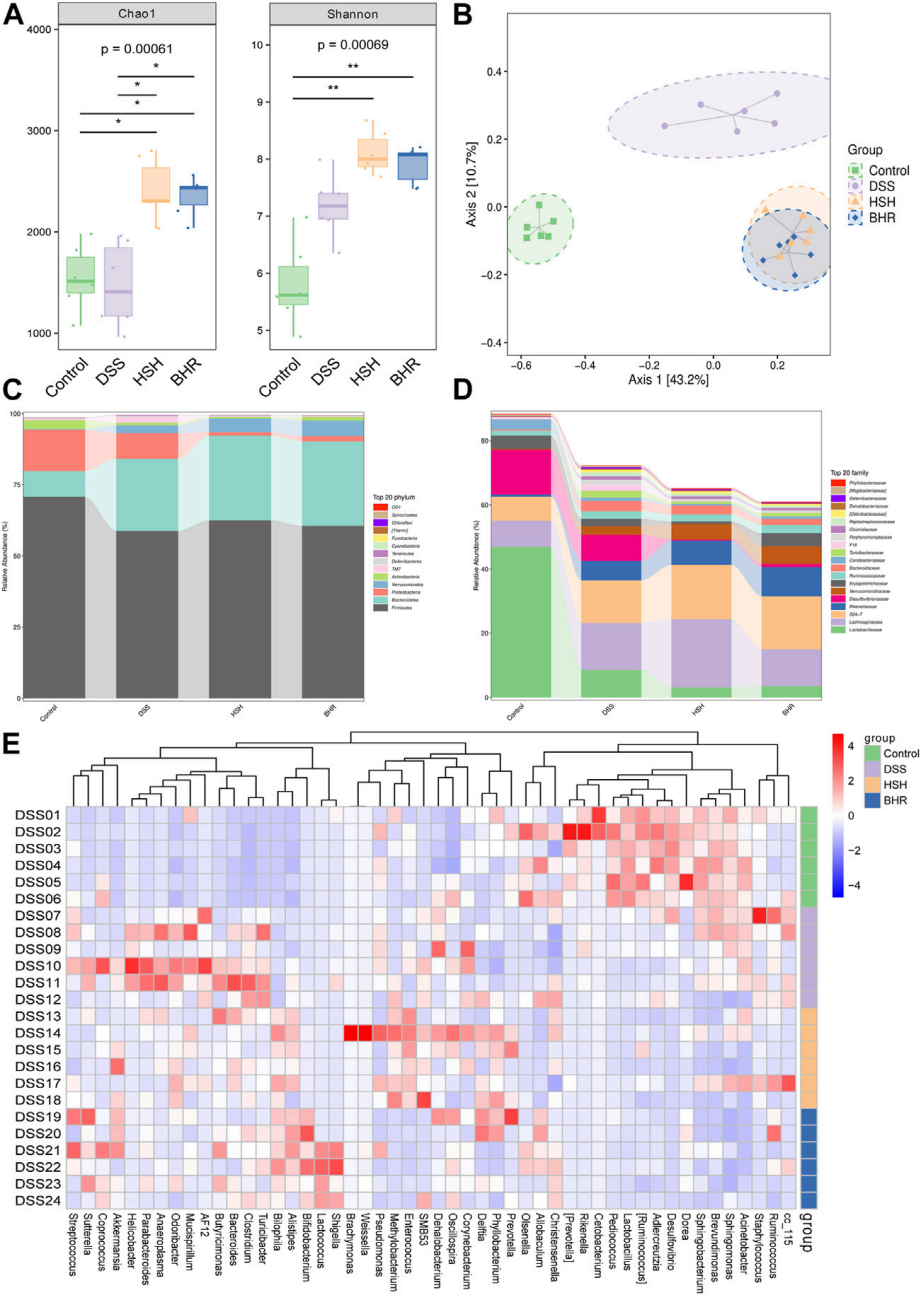
Alterations in diversity and composition of gut microbiota in DSS-treated mice with HSH and BHR administration. **(A)** *α* diversity of gut microbiota (Chao1 and shannon index). **(B)** Partial Least Squares Discrimination Analysis (PLS-DA) of *ß* diversity. **(C)** Alteration of microbial composition at phylum levels. **(D)** Alteration of microbial composition at family levels. **(E)** Heatmap of significantly changed genus in the four groups.

The bacterial communities were explored by 16S rDNA genetic augmentation (V4-V5 region) and sequencing. The evaluation of the alpha diversity is shown in Figure 5A. HSH and BHR could increase the diversity of the gut bacteria, as revealed by the Chao1 index characterizing richness and the Shannon index. The beta diversity of different groups was calculated by PCoA. The PCoA results (Figure 5B) showed that the microbial communities of the DSS group and control group were significantly separated, indicating that the microbial composition was altered dramatically and that the colitis model was established successfully. The considerable distance between the HSH/BHR group and the DSS group indicated that HSH and BHR could modulate the structure and abundance of the microbial community in colitis mice.

The top 20 most abundant bacteria at the phylum level and family level in the gut contents are presented in Figures 5C, D. Compared with the DSS group, HSH and BHR administration markedly increased the relative abundances of *Bacteroidetes* and *Verrucomicrobia* and decreased the relative abundance of *Proteobacteria* at the genus level. At the family level, HSH and BHR administration markedly increased the relative abundances of *Lachnospiraceae* and *Verrucomicrobiales* and decreased the relative abundance of *Lactobacillales*. The heatmap of bacteria at the genus level in the four groups is shown in Figure 5E. In the heatmap, HSH and BHR reversed the increases in *g_Odoribacter*, *g_Clostridium*, *g_AF12*, *g_Parabacteroides* and *g_Turicibacter* induced by DSS and reversed the decrease in *g_unclassified_Bacteria* induced by DSS. Specifically, HSH reversed the reductions in *g_unclassified_Lactobacillales* and *g_Ruminococcus* induced by DSS, and BHR reversed the declines in *g_unidentified_Coriobacteriaceae* and *g_unclassified_Firmicutes* caused by DSS treatment.

**FIGURE 6:**
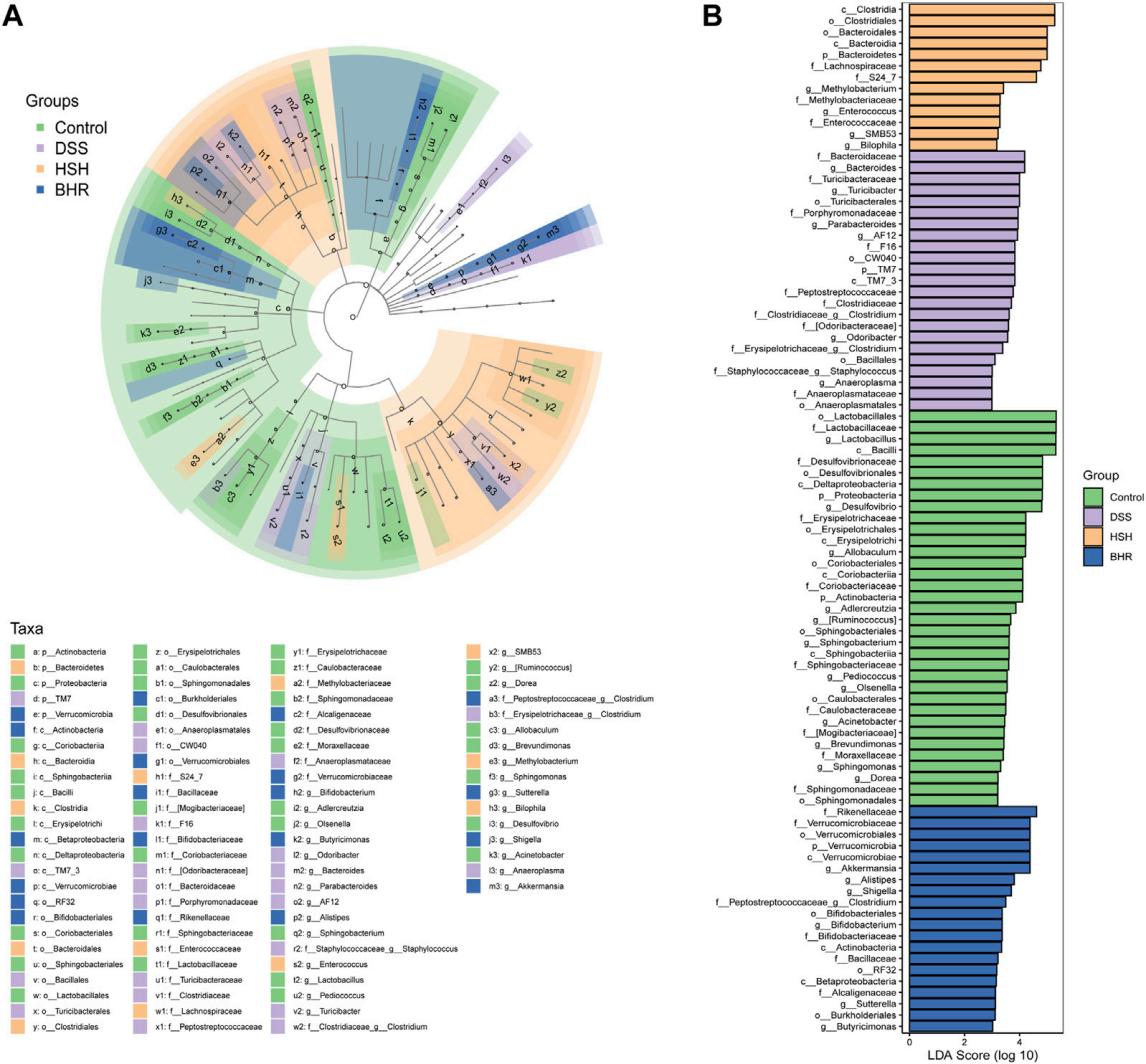
Identification of characteristic taxa with the most significant difference among four experimental groups. **(A)** Analysis of characteristic taxa among four experimental groups by linear discriminant analysis (LDA) effect size (LEfSe). **(B)** Characteristic taxa using LDA with a threshold score> 3.0. Bar length of LDA represents the impact of characteristic taxa in individual groups.

The species and relative abundance of intestinal microbiota in all groups were also analysed by LefSe. The evolutionary branch diagram showed the most differential bacteria in each group (Figure 6A). The results (Figure 6B) showed that *o_Lactobacillales*, *f_Lactobacillaceae*, *g_Lactobacillus* and *c_Bacilli* were the most differentially enriched in the control group; *f_Bacteroidaceae* and *g_Bacteroides* were the most differentially enriched in the DSS group; the HSH group had high relative abundances of *c_Clostridia* and *o_Clostridiales*; and the BHR group had high relative abundances of *f_ Rikenellaceae* and *f_ Verrucomicrobiaceae*.

### 3.6 Correlation analysis of metabolites and the gut microbiota

**FIGURE 7:**
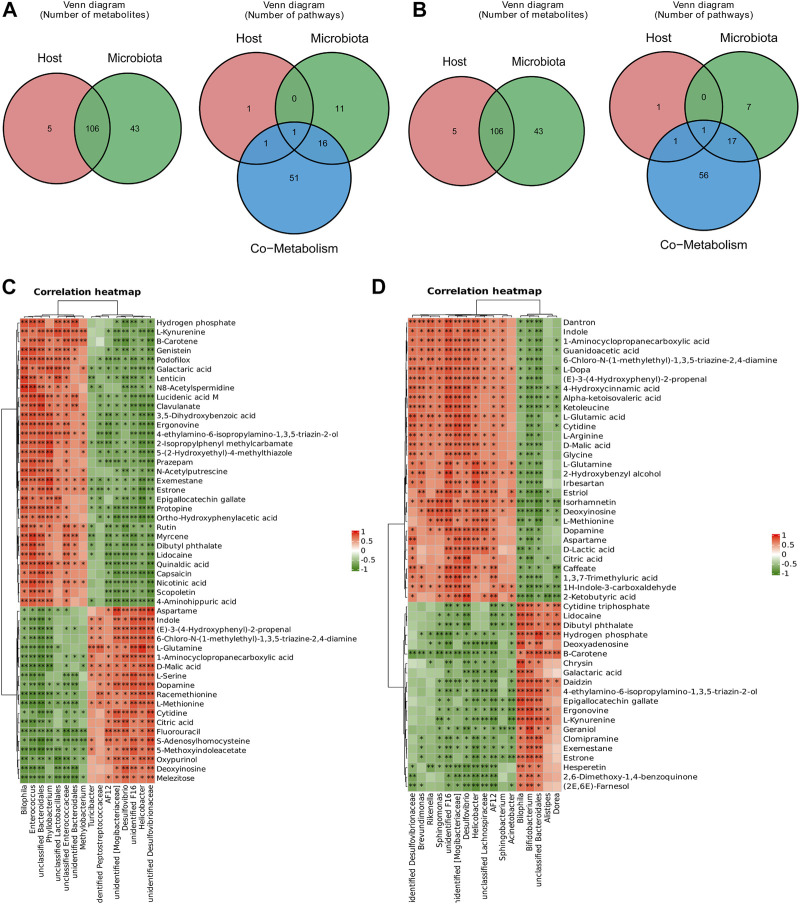
Correlation analysis between significantly changed metabolites and gut microbiota. **(A)** Significantly changed metabolites and pathways in host and microbiota in HSH treated group. **(B)** Significantly changed metabolites and pathways in host and microbiota in BHR treated group. **(C)** Correlation heatmap of significantly changed metabolites and gut microbiota in HSH treated group. **(D)** Correlation heatmap of significantly changed metabolites and gut microbiota in BHR treated group.

The host and gut microbiota metabolites were analysed, and the Venn plot (Figures 7A, B) revealed the metabolites and pathways in the host and microbiota and cometabolism. A total of 106 metabolites and one pathway (tryptophan metabolism) were cometabolized by the host and microbiota in the HSH and BHR groups. To further determine the potential relationship between the gut microbiota and metabolites, we performed Spearman’s correlation analysis between the differential bacteria at the genus level and the metabolites in the gut contents (Figures 7C, D).

In the HSH-treated group, *Bilophila*, *Enterococcus*, *Methylobacterium*, and *Phyllobacterium* were positively correlated, while *Desulfovibrio*, *Helicobacter*, and *Turicibacter* were negatively correlated with L-kynurenine, nicotinic acid, and 4-aminohippuric acid, and they had the opposite relationship with L-glutamine, malic acid, dopamine, L-methionine, cytidine, citric acid, S-adenosylhomocysteine, and deoxyinosine.

In the BHR-treated group, *Brevundimonas*, *Rikenella*, *Sphingomonas*, *Desulfovibrio*, *Helicobacter*, *Sphingobacterium*, and *Acinetobacter* were positively related, and *Bilophila*, *Bifidobacterium*, *Alistipes*, and *Dorea* were negatively related to L-dopa, alpha-ketoisovaleric acid, L-glutamic acid, cytidine, L-arginine, malic acid, glycine, L-glutamine, deoxyinosine, L-methionine, dopamine, lactic acid, citric acid, and 2-ketobutyric acid. They had the opposite relationship with L-kynurenine.

## 4 Discussion

Ulcerative colitis is an inflammatory bowel disease (IBD) that causes inflammation and ulcers in the digestive tract. There is no cure for ulcerative colitis to date. Many studies have shown that polysaccharides can effectively treat UC. *A. membranaceus* polysaccharide, *Isatis tinctoria* L. root polysaccharide, *L. barbarum* polysaccharide, *P. ginseng* polysaccharide, *Polyporus umbellaru* (Pers.) Fr. polysaccharide, *Rheum palmatum* Linn. polysaccharide and *Aconitum carmichaeli* Debx. polysaccharides were reported to be effective for the treatment of UC, mainly through anti-inflammatory and immunomodulatory activities and by modulating metabolism (Feng et al., 2020; Niu et al., 2021; Fu et al., 2022; Pan et al., 2022). Moreover, they could maintain the diversity and relative stability of the gut microbiome and downstream metabolic pathways (Zhang D. et al., 2022; Fu et al., 2022; Pan et al., 2022). In this study, we found that two polysaccharides from fungi, namely, HSH and BHR, administered at a dose of 200 mg/kg (Chen et al., 2019), could effectively relieve symptoms in the DSS-induced colitis mouse model, including body weight loss, shortened colon length, and elevated DAI scores, which indicates that HSH and BHR are promising natural product candidates for treating colitis. The underlying mechanisms need to be further investigated.

Treating ulcerative colitis usually includes anti-inflammation, immune system suppression and surgery. These treatments can only reduce the signs and symptoms of the disease. Several studies have revealed the importance of the gut microbiota in the pathogenesis of colitis (Le Gall et al., 2011). There was a decrease in the abundances of the phyla *Firmicutes* and *Actinobacteria* in UC patients and an increase in the abundances of the phyla *Proteobacteria* and *Bacteroidetes* (Alam et al., 2020). Metabolites produced by bacterial metabolism indicate that an unbalanced gut microbiota leads to metabolic dysfunction and immune disorders. *Odoribacter*, *Turicibacter*, and *Clostridium* were reported to be associated with colitis and colon cancer (Liang et al., 2020; Tang et al., 2020). These results suggest that targeting the gut microbiota may be an effective treatment method for UC. The use of 5-aminosalicylic acid in treating ulcerative colitis is common in clinical practice. It is a hydrogen peroxide scavenger that can inhibit neutrophil chemotaxis and scavenge reactive oxygen species, activate peroxisome proliferator-activated receptor *γ* or block the nuclear factor-kappa B (NF-κB) pathway. Recently, it was discovered that 5-aminosalicylic acid could ameliorate DSS-induced colitis in mice by modulating the gut microbiota and bile acid metabolism (Huang et al., 2022). Probiotic bacteria are crucial in maintaining gut microbiota homeostasis, strengthening gut barrier function and host immune responses. Probiotics (*Escherichia Coli* Nissle 1917 and VSL#3) may alleviate UC owing to their association with the intestinal flora (Dhillon and Singh, 2020; Iheozor-Ejiofor et al., 2020). *Lactobacillus acidophilus* and *Clostridium butyricum* can ameliorate colitis by strengthening gut barrier function and decreasing inflammatory factors (Wang et al., 2018). Faecal microbiota transplantation could also relieve ulcerative colitis by improving the gut microbiota and serum metabolites (Yan et al., 2018). Based on these results, targeting the gut microbiota to restore gut homeostasis may be an efficient strategy for colitis. In this study, 16S rDNA sequencing was used to reveal the changes in the gut flora before and after treatment. HSH and BHR affect the alpha and beta diversity of the bacterial communities reduced by DSS. We also observed a significant increase in the abundances of *g_Odoribacter*, *g_Clostridium*, *g_AF12*, *g_Parabacteroides* and *g_Turicibacter* in the DSS-induced colitis model, and HSH and BHR showed significant reversal effects on these bacteria. It has been proposed that the structural characteristics of polysaccharides, including their molecular weight, monosaccharide composition and glycosidic bonds, are closely related to their protective effect on UC. Polysaccharides containing galactose or mannose are more effective in treating UC (Wang et al., 2022). There are subtle differences in the modulation of the gut microbiota by HSH and BHR, mainly due to the different monosaccharide compositions. These results suggested that the normalization of the gut flora by HSH and BHR may be their primary mechanism for treating experimental colitis.

It is proposed that ulcerative colitis is closely related to metabolic changes. The metabolic profiles of serum, urine, gut tissues and faecal extracts have been measured in mouse models of ulcerative colitis and patients in previous studies (Schicho et al., 2010; Le Gall et al., 2011; Zhang et al., 2013). Alterations in lipids (cholesterol, triglycerides, phospholipids and fatty acids), amino acids (tryptophan, phenylalanine, glutamic acid, glutamine, methionine, and homocysteine), ketone bodies (3-hydroxybutyrate), energy metabolites (lactate, pyruvate and metabolites in the TCA cycle), and purines and pyrimidines have been observed in ulcerative colitis (Shiomi et al., 2011; De Preter, 2015; LINS et al., 2020; Aldars-Garcia et al., 2021; Kang et al., 2022; Yu et al., 2022). In this study, we measured the metabolites in the serum, colon tissue, gut contents and faeces of the colitis mouse model using a UPLC-TOF/MS-based untargeted metabolomics method. Consistent with previous studies, we observed significant changes in aromatic amino acid (AAA) metabolism, the citrate cycle (TCA cycle), purine metabolism, and pyrimidine metabolism in the DSS model. These results suggested that metabolic alterations may play a pivotal role in the development of ulcerative colitis. Targeting metabolism may be an effective treatment for ulcerative colitis.

It is widely accepted that the metabolites produced by the gut microbiota play essential roles in maintaining health and developing diseases. Among them, the metabolites most closely related to the gut microbiota are tryptophan metabolites, short-chain fatty acids (SCFAs), and bile acids (BAs) (Belizário et al., 2018). Several metabolite classes are reported to be potentially protective in UC, including fatty acids, amino acids and their derivatives, and bile acids (Li et al., 2022). Commensal microbiota-dependent AAA catabolism within the gastrointestinal tract exerts profound effects on host physiology, including maintaining the epithelial barrier and immune function (Agus et al., 2018; Liu et al., 2020). Metabolites of AAs are involved in cell proliferation and growth. Furthermore, IBD patients have a higher incidence of depression. Tryptophan metabolism represents a link between the gut and brain related to depression in inflammatory bowel disease (Gao et al., 2020; Chen et al., 2021). The host metabolites (kynurenine, kynurenic acid, serotonin, and melatonin) and bacterial metabolites (indole, indole-3-acetic acid, skatole, 2-oxindole, and tryptamine) of tryptophan exhibit activation potential for the aryl hydrocarbon receptor (AHR), which mediates the regulation of intestinal immunity (Dong et al., 2020; Wrzosek et al., 2021). Targeting AAA metabolism, especially tryptophan metabolism, is reported to be effective in treating colitis (Gao et al., 2018). It has been reported that supplements with *Akkermansia muciniphila* (Gu et al., 2021), deletion of ghrelin (Tuchaai et al., 2022), palmatine (Zhang et al., 2018) and polysaccharides derived from Shenling Baizhu San (Lv et al., 2022) could ameliorate colitis, mainly by suppressing tryptophan metabolism. Similar to other polysaccharides, we found that HSH and BHR have significant modulatory effects on AAA metabolism, especially tryptophan metabolism, and thus may modulate AHR and improve immunity, ultimately achieving their therapeutic effect on colitis.

The main metabolic pathways involved in immunometabolism are glycolysis, the TCA cycle, the pentose phosphate pathway, fatty acid oxidation and synthesis, and amino acid metabolism (Belizário et al., 2018; Pålsson-McDermott and O’Neill, 2020). Glycolysis is a relatively inefficient way to generate energy. It is the source of intermediate molecules for other pathways, including the pentose phosphate pathway and amino acid and fatty acid metabolism pathways, which are particularly relevant for proliferating T cells. The TCA cycle takes place in mitochondria. It activates the mitochondrial electron transport chain and produces ROS, thus activating T cells. ROS are essential during the innate immune response and inflammation and are often targeted by pathogenic bacteria (Saint-Georges-Chaumet and Edeas, 2016). Finally, the microbiota releases metabolites that can directly interfere with the mitochondrial respiratory chain and ATP production. The TCA intermediate succinate is traditionally considered an extracellular danger signal in the host (Saint-Georges-Chaumet and Edeas, 2016). Additionally, it is a primary cross-feeding metabolite among the gut resident microbes (Fernández-Veledo and Vendrell, 2019). Hippurate may be a biomarker candidate in IBD patients that was correlated with the presence of Clostridia in the gut (Storr et al., 2013). HSH and BHR both changed the metabolites in the TCA cycle, and the metabolites were related to the gut flora in the gut contents. These results suggested that HSH and BHR have a noticeable modulatory effect on the TCA cycle and energy metabolism, which may result from the regulation of the gut microbiota.

A previous study of untargeted faecal metabolomics results reflecting the functional readout of the gut microbiota showed that the predominant metabolic disturbance of the microbiota pointed to purine metabolism and pyrimidine metabolism. Metabolomics data also revealed that more metabolites were enriched in the purine metabolism pathway in the colonic and caecal epithelium of antibiotic-treated mice. Purine and pyrimidine metabolism-related metabolites were affected in the liver of the colitis mouse model (Kim et al., 2020). The altered purine metabolism resulted in an increase in the uric acid concentration. These metabolites are associated with the synthesis of nucleotides and are known to oppose inflammation and DNA damage. Rhein and luteolin alleviate inflammation and modulate the gut microbiota and purine and pyrimidine metabolism in the ulcerative colitis model (Wu et al., 2020; Li B. et al., 2021). In our study, HSH and BHR both changed the metabolites in purine and pyrimidine metabolism, and the metabolites were related to the gut flora in the gut contents; these results suggested that HSH and BHR have apparent modulation effects on purine and pyrimidine metabolism; this is probably from the regulation of the gut microbiota.

Some limitations remain in our study (Heinrich et al., 2020). First, only one dose of the two polysaccharides was administered. This is because our study is an early-stage exploratory study, and we comply with the 4R rules (reduce, refine, replace and responsibility) principle of animal welfare. Second, due to the limitations of LC-QTOF/MS, the further use of LC-MS/MS is essential to quantitate the differential metabolites accurately. Third, a mechanistic study should be performed to reveal the changes in metabolic pathways. Finally, the influence of specific gut bacterial species on the therapeutic effect of HSH and BHR needs to be further elucidated.

## 5 Conclusion

In conclusion, our study demonstrated that HSH and BHR could ameliorate colitis, and their therapeutic effects are associated with the ability to modulate systemic metabolism and gut microbiota composition. Untargeted metabolomics showed that HSH and BHR could significantly modulate the metabolites involved in AAA metabolism, the TCA cycle, purine metabolism and pyrimidine metabolism. Specifically, 16S rDNA-based gut microbiota analysis showed that HSH and BHR can alter the gut microbiota composition by increasing the abundances of *c_Clostridia* and *o_Clostridiales*, and *I. obliquus* polysaccharides increased the abundances of *f_Rikenellaceae* and *f_Verrucomicrobiaceae*. To our knowledge, this is the first comprehensive description of the effects of HSH and BHR on systemic metabolism and their relationship with gut microbiota changes in a DSS-induced mouse colitis model. This study adds to our understanding of the mechanism of polysaccharides in treating colitis and provides a new potentially effective treatment target. The separation of different polysaccharide fractions and their mechanism and structure-bioactivity relationship will be further studied.

## Data Availability

The data presented in the study are deposited in the metabolights repository, accession number MTBLS7828.

## Supplementary material

SUPPLEMENTARY FIGURE S1 The process of animal administration.

SUPPLEMENTARY FIGURE S2 Typical LC-QTOF/MS chromatograms of serum **(A)**, colon **(B)**, gut content **(C)** and faeces **(D)** in positive mode.

SUPPLEMENTARY FIGURE S3 Typical LC-QTOF/MS chromatograms of serum **(A)**, colon **(B)**, gut content **(C)** and faeces **(D)** in negative mode.

SUPPLEMENTARY FIGURE S4 The overall modulation effect of HSH and BHR on DSS-induced colitis mouse model. Scores plots of positive mode **(A)** and negative mode **(B)** of the PCA model of mouse serum from 4 groups. Scores plots of positive mode **(C)** and negative mode **(D)** of PCA model of mouse colon from 4 groups. Scores plots of positive mode **(E)** and negative mode **(F)** of PCA model of mouse gut content from 4 groups. Scores plots of positive mode **(G)** and negative mode **(H)** of PCA model of mouse faeces from 4 groups.

SUPPLEMENTARY FIGURE S5 Significantly changed metabolites in the serum of HSH and BHR treated DSS-induced colitis mouse model. The volcano plots of the DSS group compared with the Control group in positive and negative modes **(A,B)**. The volcano plots of the HSH group compared with the DSS group in positive and negative modes **(C,D)**. The volcano plots of the BHR group compared with the DSS group in positive and negative modes **(E,F)**. **(G)** Heatmap of differential metabolites of each group in positive mode. **(H)** Heatmap of differential metabolites of each group in negative mode.

SUPPLEMENTARY FIGURE S6 Significantly changed metabolites in the colon of HSH and BHR treated DSS-induced colitis mouse model. The volcano plots of the DSS group compared with the Control group in positive and negative modes **(A,B)**. The volcano plots of the HSH group compared with the DSS group in positive and negative modes **(C,D)**. The volcano plots of the BHR group compared with the DSS group in positive and negative modes **(E,F)**. **(G)** Heatmap of differential metabolites of each group in positive mode. **(H)** Heatmap of differential metabolites of each group in negative mode.

SUPPLEMENTARY FIGURE S7 Significantly changed metabolites in the gut content of HSH and BHR treated DSS-induced colitis mouse model. The volcano plots of the DSS group compared with the Control group in positive and negative modes **(A,B)**. The volcano plots of the HSH group compared with the DSS group in positive and negative modes **(C,D)**. The volcano plots of the BHR group compared with the DSS group in positive and negative modes **(E,F)**. **(G)** Heatmap of differential metabolites of each group in positive mode. **(H)** Heatmap of differential metabolites of each group in negative mode.

SUPPLEMENTARY FIGURE S8 Significantly changed metabolites in the faeces of HSH and BHR treated DSS-induced colitis mouse model. The volcano plots of the DSS group compared with the Control group in positive and negative modes **(A,B)**. The volcano plots of the HSH group compared with the DSS group in positive and negative modes **(C,D)**. The volcano plots of the BHR group compared with the DSS group in positive and negative modes **(E,F)**. **(G)** Heatmap of differential metabolites of each group in positive mode. **(H)** Heatmap of differential metabolites of each group in negative mode.

SUPPLEMENTARY FIGURE S9 The common changed pathways in serum, colon, gut content and faeces after being treated with HSH and BHR.
