# Supplementary figures and images for: Mushroom polysaccharides from Grifola frondosa (Dicks.) Gray and Inonotus obliquus (Fr.) Pilat ameliorated dextran sulfate sodium-induced colitis in mice by global modulation of systemic metabolism and the gut microbiota
Source: Front Pharmacol. 2023 Jun 7;14:1172963. doi: 10.3389/fphar.2023.1172963 (PMC10282762; doi:10.3389/fphar.2023.1172963)

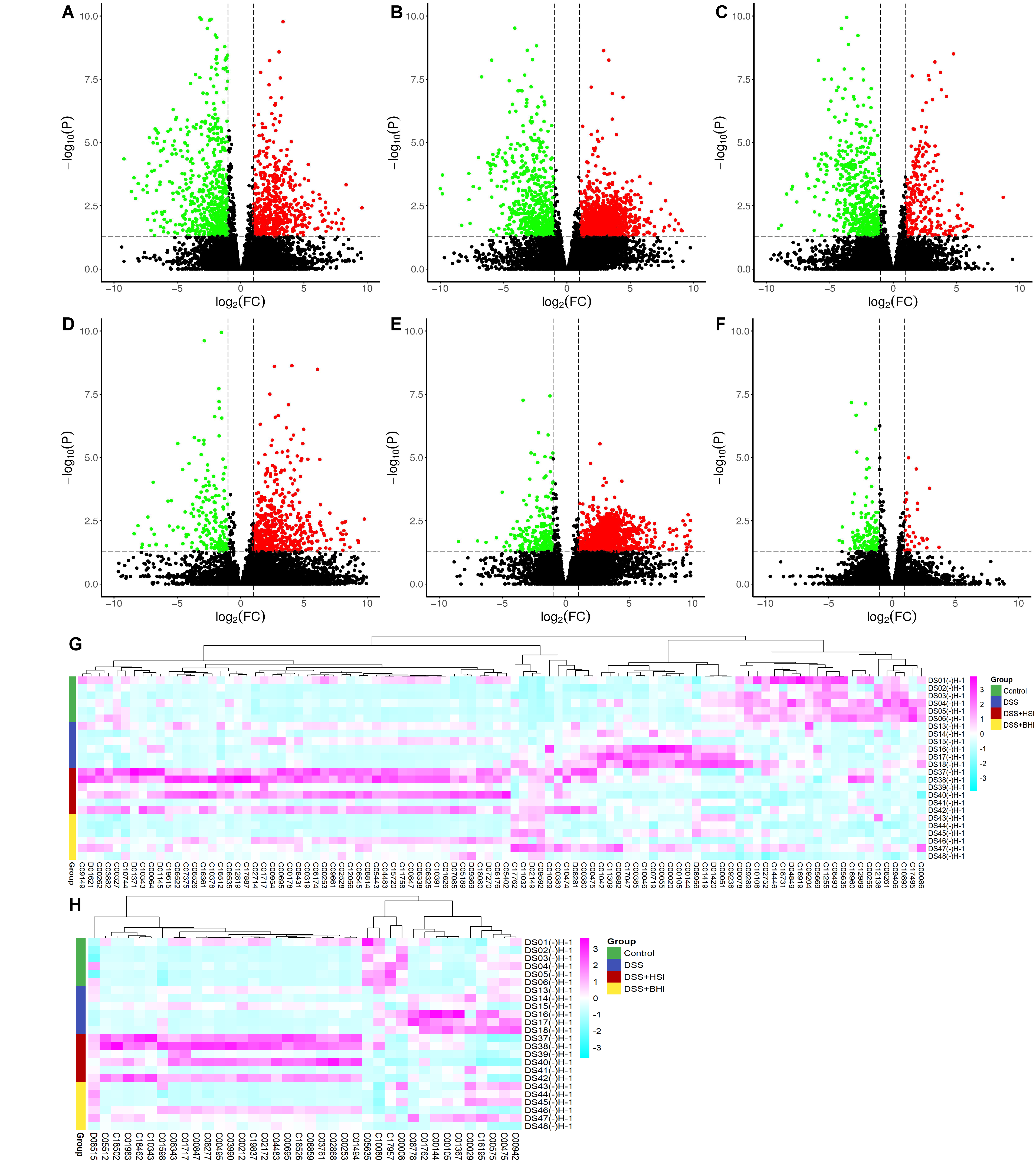

Supplement: Supplementary file 1 [file Image6.TIF]

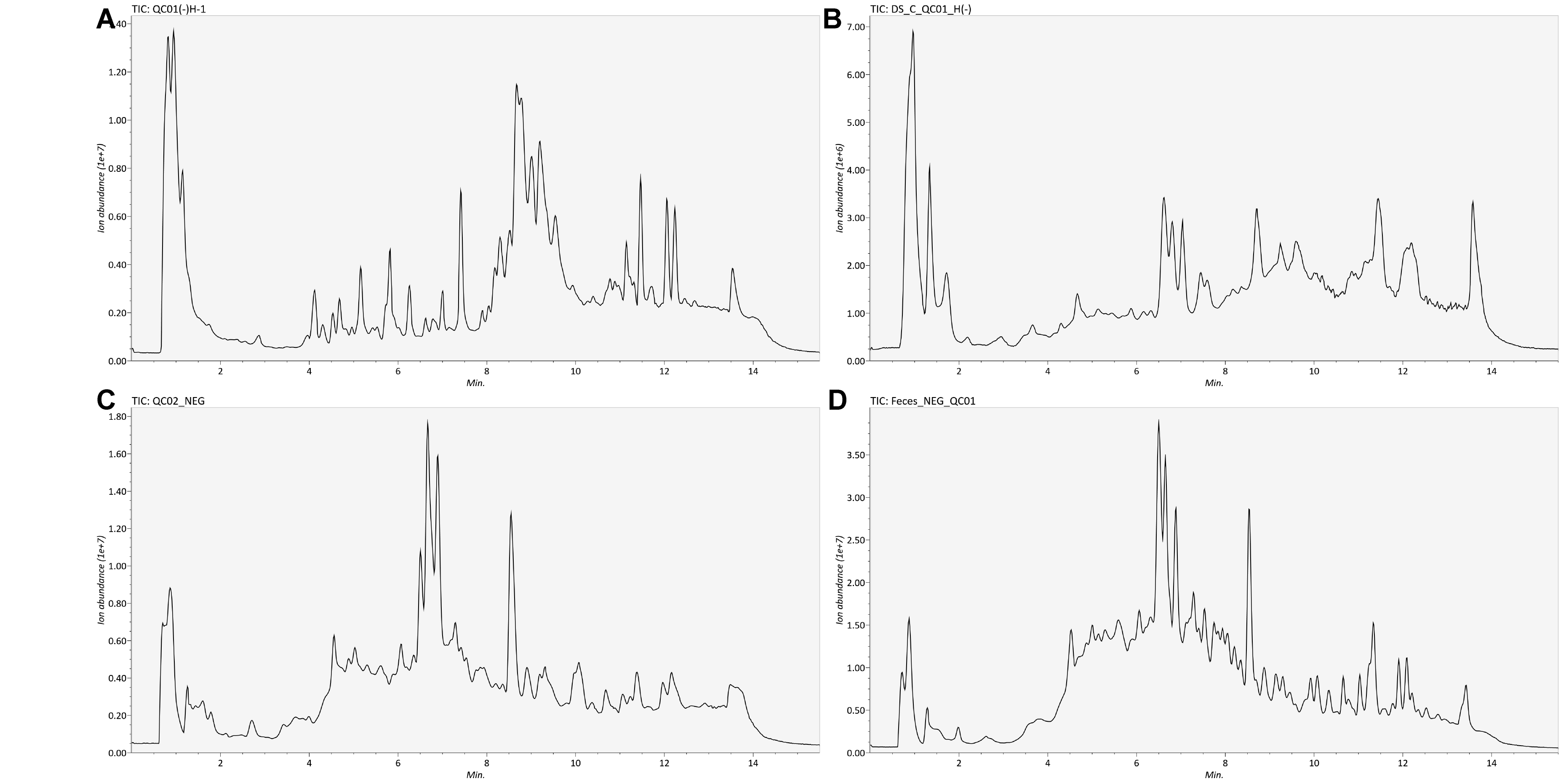

Supplement: Supplementary file 2 [file Image3.TIF]

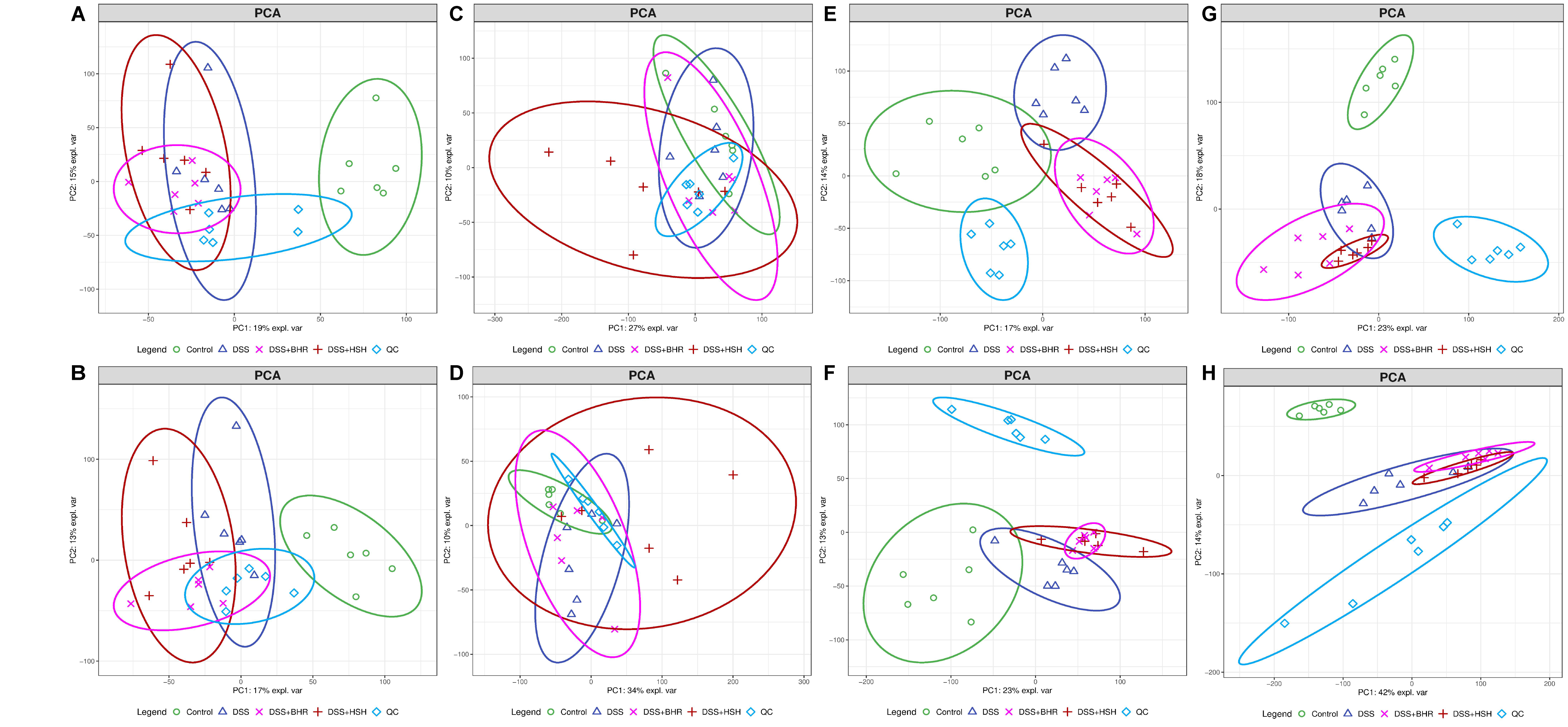

Supplement: Supplementary file 3 [file Image4.TIF]

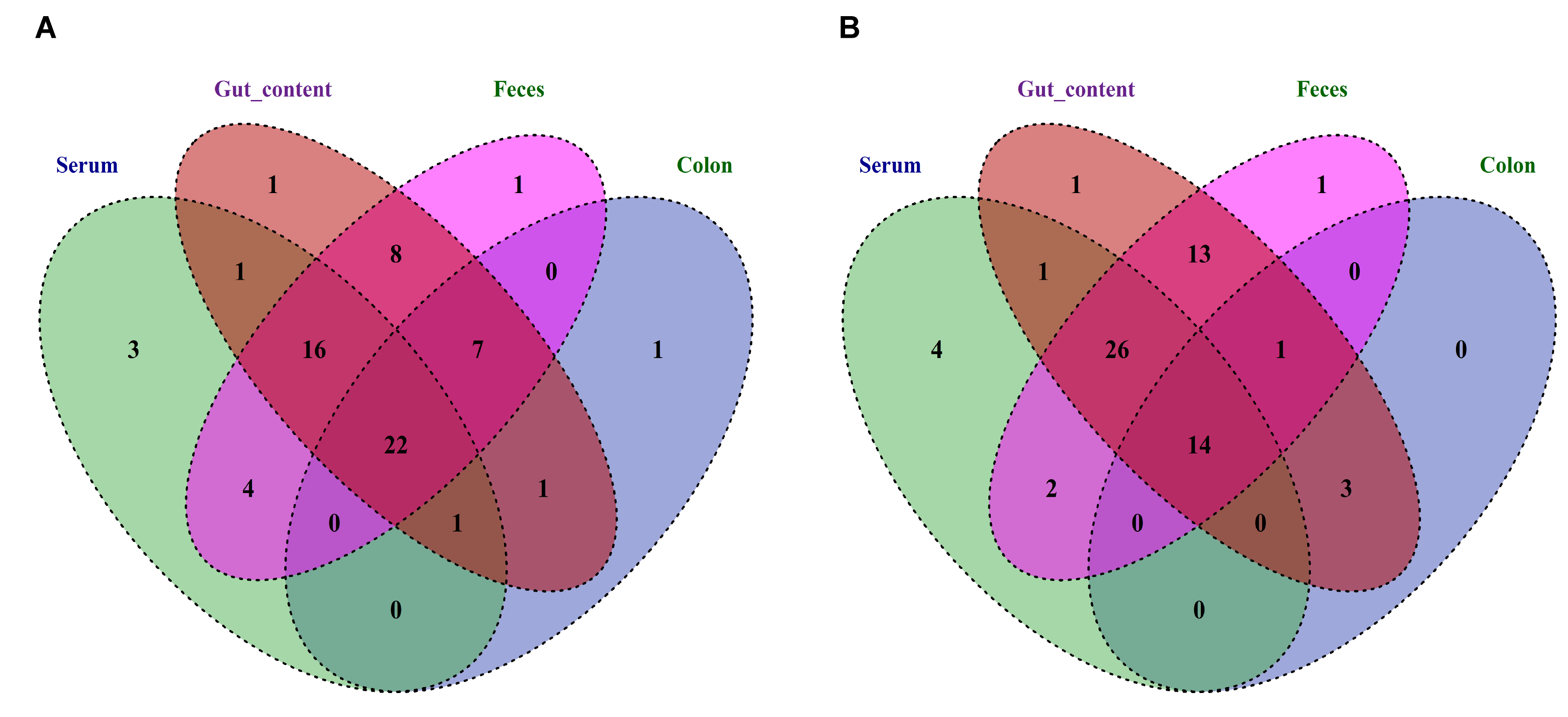

Supplement: Supplementary file 4 [file Image9.TIF]

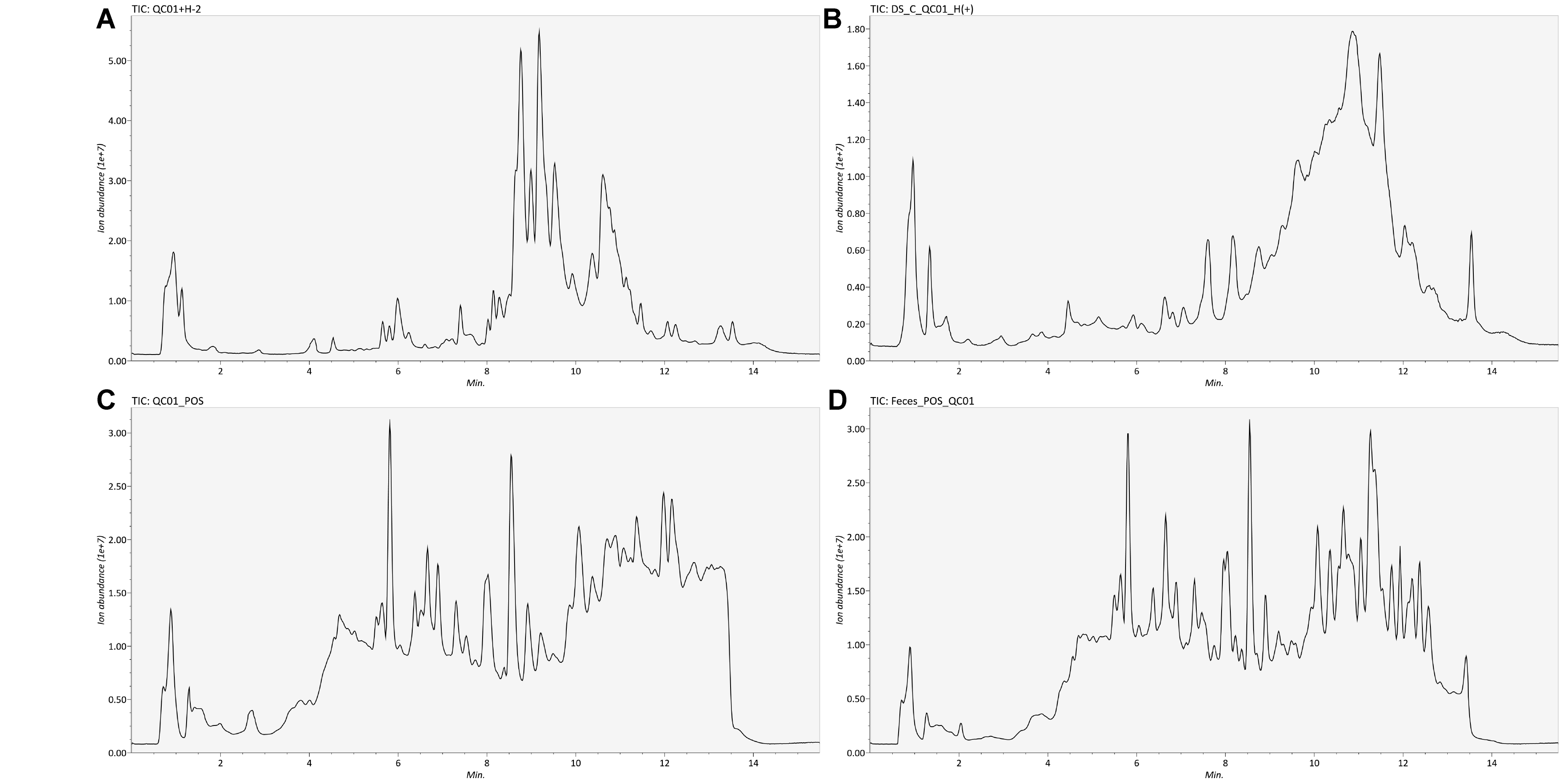

Supplement: Supplementary file 5 [file Image2.TIF]

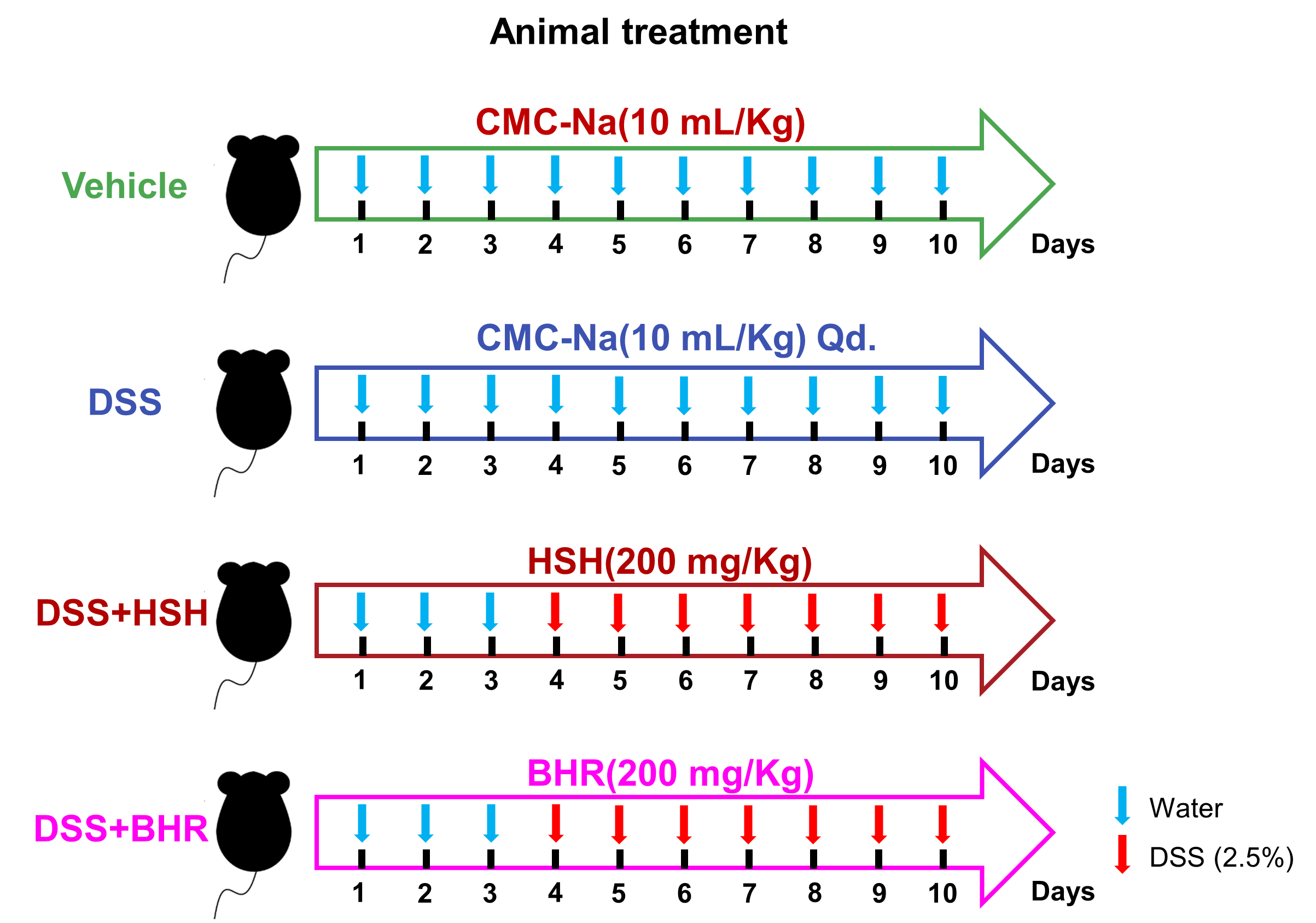

Supplement: Supplementary file 6 [file Image1.TIF]

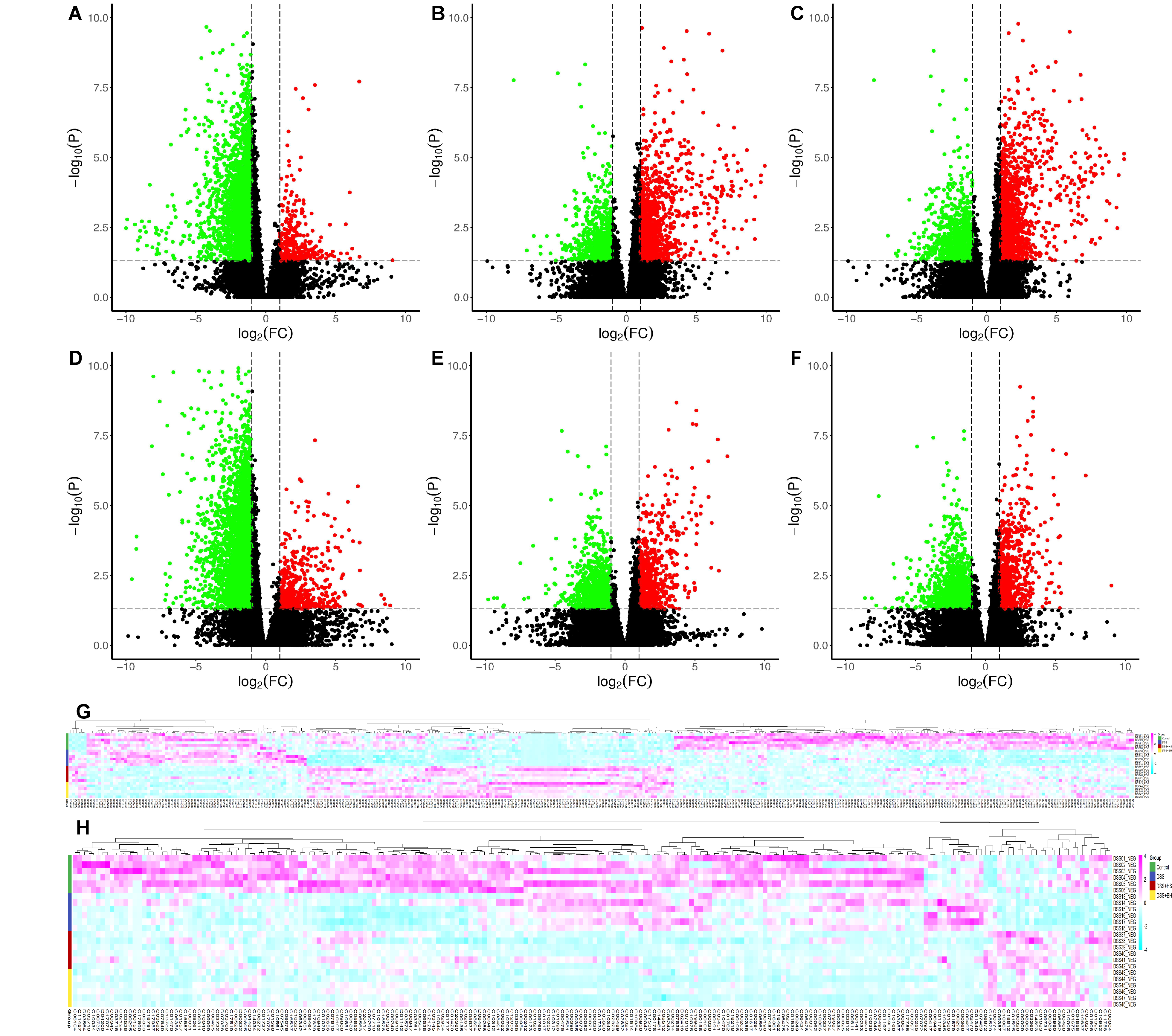

Supplement: Supplementary file 7 [file Image7.TIF]

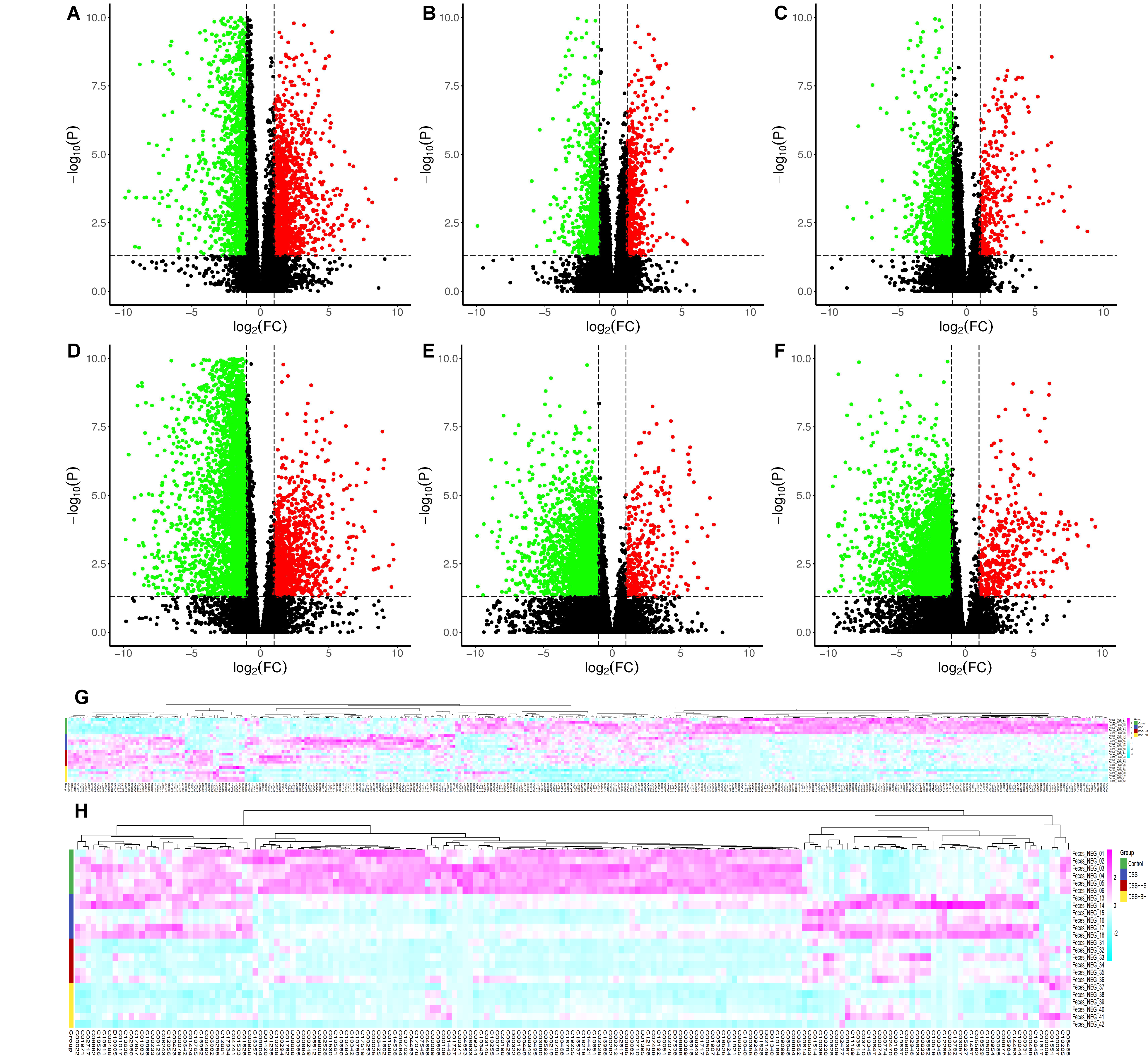

Supplement: Supplementary file 8 [file Image8.TIF]

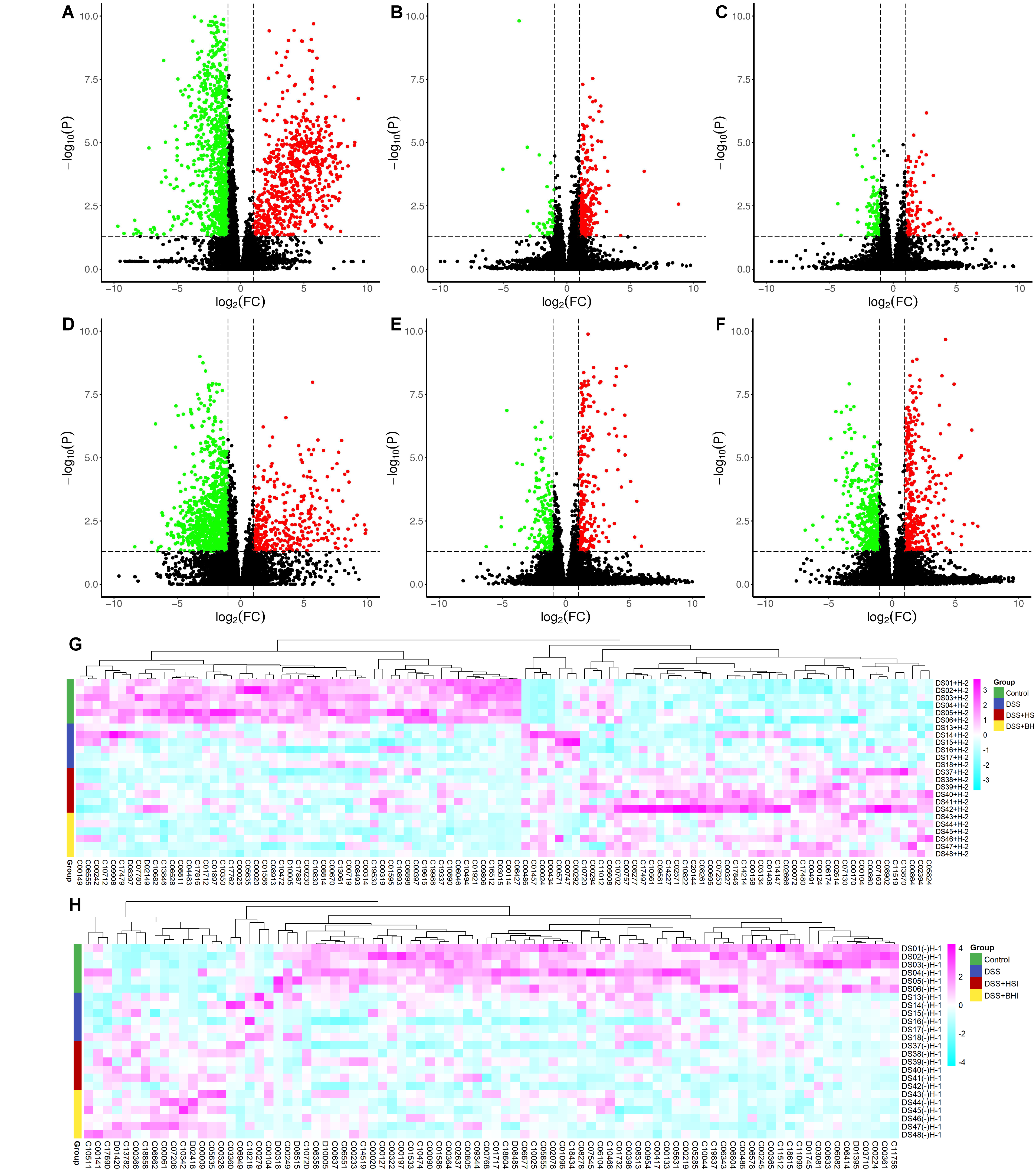

Supplement: Supplementary file 9 [file Image5.TIF]
